# An Epidemic of Three Days Fever on Board the “Bien-Hoa”

**Published:** 1918-11

**Authors:** 


***An Epidemic of Three Days Fever on Board the "Bien-Hoa"***, By the Doctors JOLY and BARIL. *Archives de Médecine et Pharmacie Navales.* October, 1918.

1. *The Epidemic on Board.*

*Importance and Extension of the Epidemic.* The disease broke out on May 11, 1918, the ship having sailed from Salonika two days before — no new case appeared after May 22.

On the whole, 107 cases were reported, including 40 per cent. of the crew, and 50 per cent. of the officers on board.

*Onset.* Generally very sudden. Marked by malaise and general fatigue lasting from one to two hours.

*Subjective Symptoms.* Intense headache and pains in the eyes, together with violent pains in the back of the neck, in the lumbar region and sometimes in the abdomen. A slight cough was usual. Very marked muscular asthenia, no appetite at all, and sometimes a slight nauseous condition.

*Objective Symptoms.* The temperature rose nearly always above $40^{0}$ C., was constant for two days and on the third day the fever subsided in slight lysis — this proved remarkably constant in all cases.

The face was generally ashen, the conjunctivae injected, the pupils slightly dilated. The tongue was covered with an abundant thick, white coating. The salivation appeared somewhat difficult.

The pharynx was congested to one-half of the soft palate. A slight tracheitis was usually present. Twice only could faint râles of congestion of the lungs be perceived, together with the disparition of the vesicular murmur, but with no change in the percussion note.

Sometimes a slight constipation existed. No change in liver and spleen — no icterus — no cardiac disturbances, only a slight tachycardia in some cases. Low arterial pressure. Kidneys functioned normally; no albumin in the urine.

*Ending of the Disease.* Two or four days of convalescence were generally necessary for complete recovery — in no cases did serious complications threaten.

*Treatment.* Two methods of treatment have been tested: in one of these, the patients were given quinine and aspirin, with no results.

In the other, the following potion was used and proved helpful to the patients, soothing their painful symptoms, but having no effect upon the fever, nor upon the general course of the disease.

| | |
|---|---|
| Sodium Salicylate . . . . . . . . . . . | 2 grammes. |
| Tincture of aconite. . . . . . . . . . . | X to XX drops. |
| Belladonna syrup . . . . . . . . . . . . | 10 grammes. |
| Aqua distillata, q. s. . . . . . . . . . | 150 grammes. |

The patients were given only fluids: pure water at first; milk and broth, later on. Tonics were often resorted to during convalescence.

*Diagnosis.* The epidemic broke out during a sudden change from fine to cold and bad weather, thus suggesting that the disease was simply due to severe colds. But this cannot be admitted, because of the general spreading of the disease, even as the weather became very fine again.

The possibility of *malaria* was discussed and rejected, and so was the diagnosis of *influenza.*

Some of the characteristics of the disease resemble those of *dengue* — but there never occurred any cutaneous exanthemas, nor any remission followed by relapse, so characteristic of dengue. However, the disease discussed in this paper presents all features of a variety of dengue, known as the "Mediterranean dengue".

*Prognosis.* The "three days fever", as met with by the authors, proved a slight disease. Recovery occurred in all cases, completely and rapidly. But it was responsible for a lessened resistance of the whole organism, and possibly also the reawakening of old pulmonary lesions. The " three days fever " coexisted in some patients with other diseases, but even then the characteristic features did not happen to be changed.

*Etiology.* The spreading of the disease occurred on board; all parts of the ship were concerned in it. Neither do the food, nor the drink, nor the process of general hygiene on board seem to be responsible for the epidemic.

2. *Epidemic Outside the Board.*

The " three days fever " extended at the same time all around the Mediterranean sea, presenting the same characters everywhere; but often with very severe complications (pulmonary congestions, pleurisy, gastro-intestinal disturbances).

3. *The Disease Previous to the Epidemic.*

The disease is not a new one. The navy medical officers know it well — it is really the "Mediterranean dengue", but spreading now widely, far away from its original focus.

*Prophylaxis.* The pathogenic agent of the disease has not been discovered as yet. Researches in the blood have always proved negative.

It seems that the small insect called "phlébostome" plays an active part in the spreading of the disease, perhaps just as mosquitoes do for malaria. But the atmosphere has also some influence, since the disease spreads most actively in badly ventilated spaces.

The authors consider that the spreading of the "three days fever" may be due to the following causes:

1) Inoculation by bites of phlébostomes.

2) Diffusion by means of flies or the atmosphere, of dried infective germs coming from the patients; feces — perhaps also convalescents remain for a long time carriers of pathogenic germs.

3) Contamination of food and drinks.

